# Human TRIM Gene Expression in Response to Interferons

**DOI:** 10.1371/journal.pone.0004894

**Published:** 2009-03-17

**Authors:** Laetitia Carthagena, Anna Bergamaschi, Joseph M. Luna, Annie David, Pradeep D. Uchil, Florence Margottin-Goguet, Walther Mothes, Uriel Hazan, Catherine Transy, Gianfranco Pancino, Sébastien Nisole

**Affiliations:** 1 Institut Cochin, Université Paris Descartes, CNRS (UMR 8104), Département des Maladies Infectieuses, Paris, France; 2 INSERM U567, Paris, France; 3 Unité de Régulation des Infections Rétrovirales, Institut Pasteur, Paris, France; 4 Section of Microbial Pathogenesis, Yale University School of Medicine, New Haven, Connecticut, United States of America; 5 Université Paris Diderot-Paris 7, UFR des Sciences du Vivant, Paris, France; University of Toronto, Canada

## Abstract

**Background:**

Tripartite motif (TRIM) proteins constitute a family of proteins that share a conserved tripartite architecture. The recent discovery of the anti-HIV activity of TRIM5α in primate cells has stimulated much interest in the potential role of TRIM proteins in antiviral activities and innate immunity.

**Principal Findings:**

To test if TRIM genes are up-regulated during antiviral immune responses, we performed a systematic analysis of TRIM gene expression in human primary lymphocytes and monocyte-derived macrophages in response to interferons (IFNs, type I and II) or following FcγR-mediated activation of macrophages. We found that 27 of the 72 human TRIM genes are sensitive to IFN. Our analysis identifies 9 additional TRIM genes that are up-regulated by IFNs, among which only 3 have previously been found to display an antiviral activity. Also, we found 2 TRIM proteins, TRIM9 and 54, to be specifically up-regulated in FcγR-activated macrophages.

**Conclusions:**

Our results present the first comprehensive TRIM gene expression analysis in primary human immune cells, and suggest the involvement of additional TRIM proteins in regulating host antiviral activities.

## Introduction

**Figure 1:**
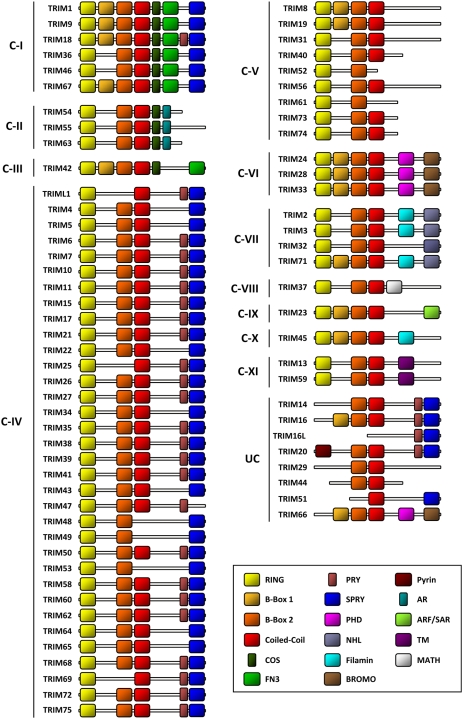
Human TRIM proteins. Classification of human TRIM proteins based on the nature of their C-terminal domains(s) as defined by Short and Cox [54] and modified by Ozato et al. [3]. The TRIM protein family is composed of 11 sub-families, from C-I to C-XI, whereas some TRIM proteins remain unclassified (UC), since they do not have a RING finger domain as “true” TRIM proteins. NHL, NHL repeats; COS, COS box motif; FN3, fibronectin type III motif; PHD, plant homeodomain; BROMO, bromodomain; MATH, meprin and TRAF homology domain; TM, transmembrane domain; AR, acid-rich region.

Tripartite motif (TRIM) proteins constitute a protein family based on a conserved domain architecture (known as RBCC) that is characterized by a RING finger domain, one or two B-box domains, a Coiled-coil domain and a variable C-terminus [1] (Figure 1). Despite their common domain architecture, TRIM proteins are implicated in a variety of cellular functions, including differentiation, apoptosis and immunity [1]. Interestingly, an increasing number of TRIM proteins have been found to display antiviral activities or are known to be involved in processes associated with innate immunity [2], [3]. TRIM5α is responsible for a species-specific post-entry restriction of diverse retroviruses, including N-MLV and HIV-1, in primate cells [4], [5], [6], [7], [8], whereas TRIM1/MID2 also displays an anti-retroviral activity which affects specifically N-MLV infection [8]. TRIM22, also known as Staf50, has been shown to inhibit HIV-1 replication, although it is still unclear at what step the block occurs [9], [10], [11]. TRIM28 restricts MLV LTR-driven transcription in murine embryonic cells [12]. Furthermore, the inhibition of a wide range of RNA and DNA viruses by TRIM19/PML has been reported [13]. The most extensive screen performed to date showed that several TRIM proteins, including TRIM11, TRIM31 and TRIM62, can interfere with various stages of MLV or HIV-1 replication [14]. Finally, TRIM25 has been shown to control RIG-I-mediated antiviral activity through its E3 ubiquitin ligase activity [15].

In one approach aimed to identify members of TRIM family with potential antiviral activity, Harmit Malik and colleagues sought TRIM proteins that have been under positive selection throughout evolution suggesting that they directly interface with ever evolving pathogens. Among these proteins are TRIM5 and TRIM22 [16], [17]. In an alternative approach, the identification of TRIM proteins up-regulated in response to interferons (IFNs) may pinpoint TRIM proteins with antiviral activities. IFNs are the main mediators of innate immunity against viral infection, by up-regulating the expression of many antiviral effectors within cells. Three classes of IFN have been identified, designated types I to III, and classified on the basis of the receptor complex they signal through, and their biological activities. Type I IFNs are a vast group of cytokines produced by most cells upon viral infection and trigger a signaling cascade that leads to the induction of many genes that control virus replication and spreading. Type I IFNs consist of multiple alpha interferon (IFN-α) subtypes and only one isoform of IFN-β, IFN-ω, IFN-ε or IFN-κ. Type II IFN only comprises one member, IFN-γ, and is produced exclusively by subsets of activated T lymphocytes and NK cells. The more recently described type III IFNs include three IFN-λ gene products. So far, little is known about the type III IFNs, although they are known to regulate the antiviral response and have been proposed to be the ancestral type I IFNs [18], [19].

Strikingly, most of the TRIM proteins implicated in antiviral response, including TRIM5 [20], [21], [22], TRIM19/PML [23], [24], [25], TRIM20/MEFV [26], TRIM21/Ro52 [27], [28], TRIM22 [9], [10], TRIM25 [29], [30] and TRIM34 [31] have also been found to be up-regulated by IFNs. In addition, microarrays have contributed to information about the gene expression of TRIM proteins. For example, in the human fibrosarcoma cell line HT1080, TRIM19 (PML) and TRIM21 (52-kD SS-A/Ro autoantigen) were found to be induced by both type I and II IFNs, whereas TRIM22 (Staf50) expression was only up-regulated by type I (α and β) IFN [32]. Similarly, TRIM19/PML, 21 (SSA1), 22 and 25 (ZNF147) were found to be up-regulated by pegylated interferon-alpha2b in human peripheral blood cells [33]. In murine cells, a recent study of gene expression of a significant proportion of TRIM proteins and an additional microarray study provided some insight into the expression of this protein family in mouse [34], [35]. However, no comprehensive study has been performed thus far for the entire TRIM protein family. Besides IFN, ITAM-coupled receptors for the Fc region of immunoglobulins (FcRs) regulate macrophage responses to pathogens [36]. Activating FcγR signaling via ITAM motifs not only triggers signaling pathways different from those activated by IFNs, but FcγR cross-linking by IC can negatively regulate IFN-induced signaling [37], [38]. We have shown that the aggregation of FcγR by immune complexes (IC) inhibits replication of HIV-1 and related lentiviruses in human monocyte-derived macrophages [39], [40]. FcγR-mediated restriction affects early pre-integrative steps of HIV-1 replication and might be compatible with a TRIM5α-like restriction mechanism [40]. No information is available on the regulation of TRIM protein expression following FcγR engagement.

Here we report a systematic study of the expression of all TRIM genes and their sensitivity to type I IFN, type II IFN, and FcγR signaling in human primary lymphocytes and macrophages. We decided not to include type III IFNs in our study since, despite binding to distinct receptors from those of type I IFNs, type III IFNs induce antiviral activity using the same signaling pathway, the same IFN-stimulated response elements (ISREs), and lead to the induction of almost the same IFN-stimulated genes (ISGs) than type I (α and β) IFNs [18], [41]. We applied quantitative RT-PCR arrays to quantify the expression of the 72 human TRIM genes in treated or untreated cells. Although some of these genes cannot be considered as “real” TRIM genes since they encode proteins that do not contain an intact RBCC architecture, we decided to include them in our study, as they probably derive from a common ancestor gene.

## Materials and Methods

### Monocyte derived macrophages (MDM) and peripheral blood lymphocytes (PBL)

Blood samples were obtained through the French blood bank (Etablissement Français du Sang, EFS) in the setting of the EFS-Institut Pasteur Convention. A written informed consent was obtained from each donor to use the cells for clinical research according to French laws. The study was approved by two IRBs, one for EFS, as required by French law, and one for Pasteur Institute (the Biomedical Research Committee), as required by Pasteur Institute. Human monocytes and lymphocytes were isolated from buffy coats of healthy seronegative donors (Centre de Transfusion Sanguine Ile-de-France, Rungis and Hôpital de la Pitié-Salpêtrière, Paris, France) using lymphocyte separation medium (PAA laboratories GmbH, Pasching, Austria) density gradient centrifugation and plastic adherence as previously described [40]. Non adherent cells (PBL) were frozen in 90% fetal calf serum (FCS) and 10% DMSO at −80°C until the experiment of activation. Monocytes were then differentiated into macrophages by 7 to 11 days culture in MDM medium (RPMI 1640 medium supplemented with 200 mM L-glutamine, 100 U penicillin, 100 µg streptomycin, 10 mM HEPES, 10 mM sodium pyruvate, 50 µM β-mercaptoethanol, 1% minimum essential medium vitamins, and 1% nonessential amino acids) supplemented with 15% of human AB serum in hydrophobic Teflon dishes (Lumox™, Dominique Dutcher, Brumath, France) as previously described [40]. Monocyte-derived-macrophages (MDM) were then harvested, washed and resuspended in MDM medium containing 10% heat-inactivated FCS for experiments. Purity of MDM was assessed by flow cytometry by side and forward scattering and immunofluorescent staining. Cells obtained by this method are 91–95% CD14^+^, and express CD64, CD32, and CD16 FcγRs.

One day before experiments, PBL were thawed and cultured in PBL medium (RPMI 1640 medium supplemented with 200 mM L-glutamine, 100 U penicillin, 100 µg streptomycin, 10% FCS).

PBL and MDM were seeded in 6 well plates (2×10^6^ PBL/well, 1×10^6^ MDM/well) in the presence or not of 1000 UI/ml of IFN I (Universal type I IFN, PBL Biomedical Laboratories, New Brunswick, USA)(Universal type I IFN is an hybrid alpha interferon, constructed from recombinant human IFN-α A and human IFN-α D) or IFN II (IFN-γ, Peprotech EC Ltd, London, UK). MDM stimulation with preformed immune complexes (IC) was performed as previously described [40]. Briefly, culture plates were coated with 0.1 mg/ml dinitrophenyl-conjugated bovine serum albumin (DNP-BSA) by incubation for 2 hours at 37°C, saturated with 1 mg/ml BSA in PBS, and then incubated 1 h hour at 37°C with 30 µg/ml rabbit anti-DNP antibodies (Sigma, Saint Louis, USA) to form ICs. All reagents used were LPS-free. After washing of the plates with PBS, MDM were stimulated by plating on IC-coated wells.

Eight hours after stimulation with either IFNs or IC, cells were finally washed and frozen at −80°C in the presence of 350 µl of RLT buffer/well (RNeasy Mini kit, Qiagen). RNA extractions were performed using RNeasy Mini Kit following manufacturer's instructions. Experiments were performed using PBL and MDM from 3 different donors.

### qRT-PCR array analysis

We designed custom RT^2^ Profiler PCR arrays (SABiosciences, Frederick, USA) in order to quantify simultaneously the expression of 72 human TRIM genes and 14 other human genes for control. The complete list of the 86 screened genes is shown in Table 1. Briefly, total RNA from PBL or MDM isolated from 3 donors and treated or not with IFN I, IFN II or IC were reverse transcribed using the RT^2^ PCR array first strand kit (SABiosciences). PCR were performed using the RT^2^ Realtime SYBR Green PCR mix (SABiosciences) following manufacturer's instructions on a LightCycler 480 (Roche Diagnostics, Meylan, France). Data were analyzed by the 2^−ΔΔCt^ method. Briefly, threshold cycle (Ct) values were converted to 2^−Ct^ in order to be proportional to the amount of transcripts in the samples. For comparing samples between them, 2^−ΔCt^ were calculated by normalizing the data by a housekeeping gene (HKG): 2^−ΔCt^ = 2^−Ct^(sample)/2^−Ct^(HKG). Finally, in order to compare the data from different experimental conditions, we calculated 2^−ΔΔCt^ values, which are obtained by normalizing the experimental data by reference data. For example, data from treated cells are normalized to untreated cells, according to the formula: 2^−ΔΔCt^ = 2^−ΔCt^(treated cells)/2^−ΔCt^(untreated cells). Differentially expressed genes were defined as those that changed by >2-fold. Java TreeView was used to represent data as heat map representations [42].

**Table 1:** Genes screened in the PCR array analysis.

TRIM	Official	Accession No.	Amplicon	Amplicon	TRIM	Official	Accession No.	Amplicon	Amplicon
	symbol	(Genbank)	size (bp)	position		symbol	(Genbank)	size (bp)	position
**1**	MID2	NM_012216	155	2150–2168	**45**		NM_025188	123	2311–2331
**2**		NM_015271	51	3325–3344	**46**		NM_025058	158	2184–2202
**3**		NM_006458	130	2032–2053	**47**		NM_033452	175	1304–1326
**4**		NM_033017	134	3081–3102	**48**		NM_024114	89	140–161
**5**		NM_033093	111	924–943	**49**		NM_020358	107	1245–1267
**6**		NM_058166	181	1035–1054	**50**		NM_178125	137	1197–1215
**7**		NM_033342	159	767–787	**51**	SPRYD5	NM_032681	191	605–625
**8**		NM_030912	111	1450–1468	**52**		NM_032765	191	312–332
**9**		NM_015163	93	1958–1976	**53**		XR_016180	181	1052–1071
**10**		NM_006778	181	785–807	**54**		NM_187841	161	752–771
**11**		NM_145214	158	1279–1299	**55**		NM_184087	85	662–681
**13**		NM_005798	158	389–411	**56**		NM_030961	158	459–477
**14**		NM_014788	140	559–577	**58**		NM_015431	172	948–966
**15**		NM_033229	100	2024–2048	**59**		NM_173084	114	541–561
**16**		NM_006470	148	1004–1022	**60**		NM_152620	113	266–284
**16L**		NM_001037330	125	20–43	**61**		NM_001012414	176	431–453
**17**		NM_016102	153	1216–1234	**62**		NM_018207	94	1177–1197
**18**	MID1	NM_000381	139	2214–2232	**63**		NM_032588	100	1567–1588
**19**	PML	NM_033238	67	1328–1348	**64**		XM_061890	191	1213–1233
**20**	MEFV	NM_000243	165	1906–1924	**65**		NM_173547	141	2870–2888
**21**		NM_003141	173	1137–1159	**66**		XM_084529	88	6581–6601
**22**		NM_006074	139	2293–2313	**67**		NM_001004342	185	8275–8296
**23**		NM_001656	143	3551–3574	**68**		NM_018073	180	1322–1341
**24**		NM_003852	191	2560–2581	**69**		NM_182985	99	1409–1431
**25**		NM_005082	161	965–985	**71**		NM_001039111	171	2061–2079
**26**		NM_003449	154	1576–1595	**72**		NM_001008274	132	1504–1522
**27**		NM_006510	164	2735–2754	**73**		NM_198924	121	1195–1219
**28**		NM_005762	131	1737–1755	**74**		NM_198853	123	321–341
**29**		NM_012101	84	2833–2851	**L1**		NM_178556	173	1138–1157
**30**		NM_007028	166	1723–1743		PPIA	NM_021130	191	838–861
**32**		NM_012210	179	326–345		STAT1	NM_007315	92	199–221
**33**		NM_015906	132	3370–3391		EIF2AK2	NM_002759	84	1395–1415
**34**		NM_021616	134	849–868		HIST4H4	NM_175054	92	120–141
**35**		NM_171982	190	421–439		OAS2	NM_002535	139	345–363
**36**		NM_018700	165	482–501		MX1	NM_002462	184	1883–1905
**37**		NM_015294	94	2883–2902		ADAR	NM_001111	150	3896–3918
**38**		NM_006355	172	1486–1505		APOBEC3G	NM_021822	156	1103–1124
**39**		NM_021253	131	1628–1649		APOBEC3F	NM_145298	89	4610–4630
**40**		NM_138700	105	398–418		CSF1	NM_000757	181	523–545
**41**		NM_201627	181	868–889		HPRT1	NM_000194	89	974–993
**42**		NM_152616	101	2371–2391		RPL13A	NM_012423	90	940–960
**43**		NM_138800	157	1156–1176		GAPDH	NM_002046	175	1287–1310
**44**		NM_017583	120	1097–1119		ACTB	NM_001101	191	1202–1222

### Phylogenetic analysis of human TRIM proteins

The amino-acid sequences of all human TRIM proteins were obtained from the *“HUGO Gene Nomenclature Committee at the European Bioinformatics Institute”* (http://www.genenames.org). A neighbor-joining tree was constructed with NJplot (from http://pbil.univ-lyon1.fr/software/njplot.html), on the basis of a ClustalX2 sequence alignment of all TRIM proteins deleted from their Ct domain(s) (http://www.clustal.org/), with a bootstrap trial of 1000. TRIM alignments are available from the authors by request.

### Promoter *in silico* analysis

Promoter analysis was carried out using the PROMO virtual laboratory (http://alggen.lsi.upc.es/cgi-bin/promo_v3/promo/promoinit.cgi?dirDBTF_8.3) and Genomatix MatInspector (http://www.genomatix.de/products/MatInspector/) programs for identifying putative transcription factor binding sites [43], [44], [45]. Briefly, 1 kb of DNA upstream of the predicted transcription start site for each TRIM protein along with 100 bp reading into the mRNA (−1000 bp to +100 bp) was selected from the human genome for analysis. These sequences were analyzed in PROMO and MatInspector using versions 8.3 and 7.1 of the TRANSFAC matrix library respectively. For PROMO, hits were scored for specific transcription factors based on dissimilarity values of less than 0.1 and random expectation values of less than 0.01. For MatInspector, hits were scored on matrix similarities above 0.8. Genomic positive controls consisting of promoter regions known to possess binding sites for each selected transcription factor were used to evaluate the stringency of the PROMO and MatInspector algorithms to determine significant results (STAT1, OAS2, MX1, APOBEC3G, etc). Negative controls consisting of housekeeping genes (GAPDH, ACTB) and randomized DNA sequences were used to evaluate and eliminate less stringent matrices.

## Results

**Figure 2:**
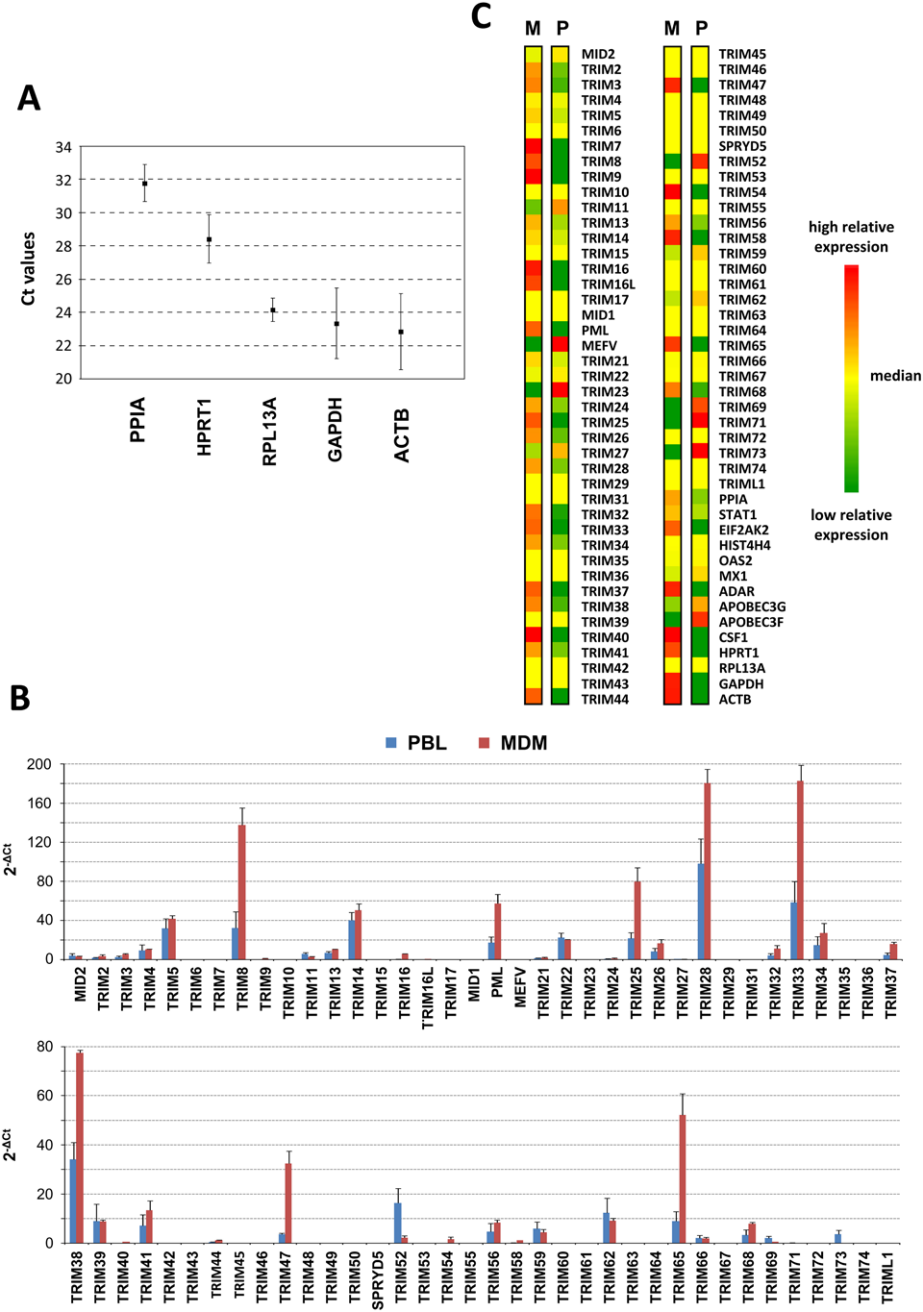
TRIM gene expression in human primary macrophages and lymphocytes. cDNA was prepared from primary macrophages (MDM) and lymphocytes (PBL) from 3 donors, as described in the Materials and Methods section. The expression of 86 genes, including 72 TRIM genes and 5 housekeeping genes, was analyzed by quantitative RT-PCR array. A. Comparison of the expression of 5 housekeeping genes in untreated MDM and PBL. The mean Ct values for each gene in untreated cells from 3 donors are shown. Error bars show standard deviation. RPL13A presented the smallest standard deviation values and was therefore selected for normalization. B. Constitutive expression of TRIM genes in MDM (M) and PBL (P). Histograms represent mean 2^−ΔCt^ values for each gene±SD. C. Relative expression of TRIM genes in MDM (M) and PBL (P). Mean 2^−ΔCt^ values were determined by subtracting RPL13A, and each sample was normalized to the median expression of each gene in both cell types. Resulting 2^−ΔΔCt^ values were represented as a heat map, using Java TreeView. Green: low relative expression; Yellow: median value (same expression in MDM and PBL); Red: high relative expression.

In order to perform a comprehensive study of TRIM gene expression in human primary lymphocytes and macrophages, unstimulated peripheral blood lymphocytes (PBL) and monocyte derived macrophages (MDM) from 3 donors were either left untreated or stimulated with type I IFN, type II IFN or immune complexes (IC, in the case of MDM only), as indicated in the Materials and Methods section. After RNA extraction and cDNA preparation, we screened 86 gene transcripts by real-time quantitative PCR. In addition to the 72 TRIM gene transcripts, we also analyzed the expression of a number of housekeeping genes to standardize the assays, such as PPIA (peptidylprolyl isomerase A, cyclophilin A), HPRT1 (hypoxanthine phosphoribosyltransferase 1), RPL13A (ribosomal protein L13a), GAPDH (glyceraldehyde-3-phosphate dehydrogenase) and ACTB (actin beta) (Table 1). We also included some genes whose expression is known to be either down-regulated by IFN, such as HIST4H4 (histone cluster 4, H4) or up-regulated, such as STAT1 (signal transducer and activator of transcription 1, 91kDa), EIF2AK2 (Homo sapiens eukaryotic translation initiation factor 2-alpha kinase 2, PKR), OAS2 (2′-5′-oligoadenylate synthetase 2, 69/71 kDa), MX1 (myxovirus resistance 1), ADAR (adenosine deaminase, RNA-specific), APOBEC3G and APOBEC3F (apolipoprotein B mRNA editing enzyme, catalytic polypeptide-like 3G and 3F) (Table 1) [32], [46]. Furthermore, we analyzed CSF1 (or M-CSF, Macrophage colony-stimulating factor 1) expression as a control for IC-mediated MDM activation, since we have previously shown that CSF1 expression is up-regulated following FcγR cross-linking [39]. Finally, we included internal controls to account for genomic DNA contamination, reverse transcription efficiency and PCR efficiency, in order to validate the screen and allow proper comparison between experiments. All data were analyzed using the 2^−ΔΔCt^ method. From the collected data, we first analyzed the basal expression of the screened genes in untreated MDM and PBL. In order to compare the basal expression of each gene in lymphocytes and MDM, we normalized our values by a housekeeping gene whose expression is as similar as possible in both cell types. Toward this end, we compared the mean Ct values for each housekeeping gene in untreated MDM and PBL. As shown in Figure 2A, RPL13A presents the smallest standard deviation values among the 5 selected housekeeping genes, demonstrating that its expression was almost identical in PBL and MDM. Therefore, we used RPL13A to compare TRIM gene expression between both cell types.

Figures 2B and 2C compare absolute (2B) or relative (2C) TRIM gene expression in human MDM and lymphocytes. Among the 72 analyzed TRIM transcripts, 27 were not detectable in either MDM or PBL (TRIM6, 10, 15, 17, 18/MID1, 29, 31, 35, 36, 42, 43, 45, 46, 48, 49, 50, 51/SPRYD5, 53, 55, 60, 61, 63, 64, 67, 72, 74 and L1) (Figure 2B). Some were specifically expressed in MDM (TRIM7, 9, 40 and 54) or in PBL (TRIM20/MEFV, 23, 71 and 73) (Figures 2B and 2C).

**Figure 3:**
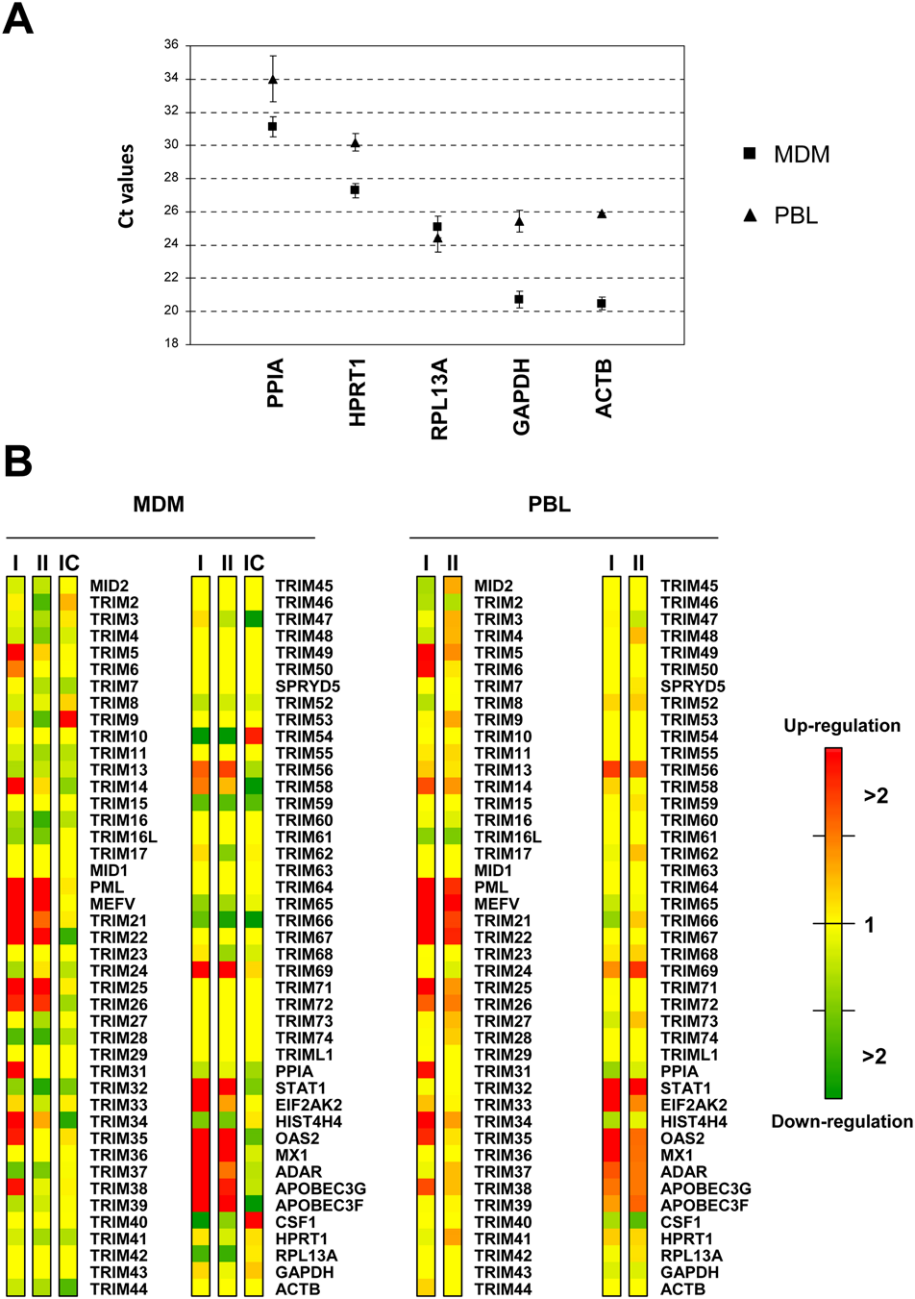
TRIM gene expression in response to IFN or immune complex. Primary macrophages (MDM) or lymphocytes (PBL) from 3 donors were left untreated or were treated with type I IFN, type II IFN or preformed immune complexes (IC, in the case of MDM only), as described in the Materials and Methods section. The expression of 86 genes, including 72 TRIM genes and 5 housekeeping genes, was analyzed by PCR array. A. Selection of a housekeeping gene to normalize the expression of TRIM genes in untreated Vs IFN- or IC-treated cells. The diagram shows the mean expression of 5 housekeeping genes in untreated and treated MDM (squares) or PBL (triangles). The mean Ct values for each gene in untreated cells and IFN or IC-treated cells from 3 donors are shown. Error bars show standard deviation. ACTB presented the smallest standard deviation values and was therefore selected for normalization. B. Induction of TRIM genes in primary cells treated with type I IFN (I), type II IFN (II) or IC. Mean 2^−Ct^ values for each gene in cells from 3 donors were normalized to ACTB expression to calculate 2^−ΔCt^ values. Normalization to the mean expression of each gene in untreated cells gave the 2^−ΔΔCt^ values, which were presented as a heat map using Java TreeView. Green: Down-regulation of gene expression; Yellow: No change; Red: Up-regulation of gene expression. A significant modification of gene expression was defined as a >2 down- (dark green) or up-regulation (dark red).

Next, we analyzed which genes are regulated by IFN in both cell types and by IC in MDM. As described above, we first determined the housekeeping gene that had the most stable expression in a given cell type upon IFN or IC treatment in order to normalize our results. Mean Ct values of the 5 screened housekeeping genes in untreated and treated cells with either type I or type II IFN (as well as IC in the case of MDM) were calculated, along with the corresponding standard deviation values (Figure 3A). Actin B (ACTB) expression was found to be almost identical in untreated and IFN-treated PBL and also in untreated, IFN- or IC-treated MDM and was thus selected as the most suitable gene to normalize our results in both cell types.

The regulation of each TRIM gene after normalization to ACTB expression is shown in Figure 3B. We considered gene expression variations as significant when we observed a >2-fold variation compared to untreated cells. Genes that were significantly up- or down-regulated are represented in red and green in the heat map representation, respectively (Figure 3B). As expected, we found that STAT1, EIF2AK2, OAS2, MX1, ADAR, APOBEC3G and APOBEC3F were significantly up-regulated by both type I and type II IFN [32], and that TLR4 expression was up-regulated by type I IFN in MDM [46], whereas HIST4H4 expression was down-regulated upon IFN-treatment [32]. In MDM, type I IFN up-regulated the expression of 16 TRIM genes (TRIM5, 6, 14, 19/PML, 20/MEFV, 21, 22, 25, 26, 31, 34, 35, 38, 56, 58 and 69) and down-regulated the expression of 5 (TRIM28, 37, 54, 59 and 66). Among these TRIM genes, type II IFN only induced the up-regulation of 7 genes (TRIM19/PML, 20/MEFV, 21, 22, 25, 56 and 69) but induced the down-regulation of 11 (TRIM2, 4, 9, 16, 16L, 28, 32, 37, 54, 59 and 66). In PBL, 14 TRIM genes were up-regulated by type I IFN (TRIM5, 6, 14, 19/PML, 20/MEFV, 21, 22, 25, 26, 31, 34, 35, 38 and 56) and 7 by type II IFN (TRIM19/PML, 20/MEFV, 21, 22, 26, 56 and 69) (Figure 3B). The only gene whose expression was significantly down-regulated in PBL following IFN treatment was TRIM16L (Figure 3B). Interestingly, whereas TRIM6, TRIM31 and TRIM35 transcripts were undetectable in either untreated MDM or PBL, they could be detected in type I IFN-treated cells. The expression of TRIM9 in PBL was also weakly induced by type II IFN. Similarly, both type I and type II IFN induced the expression of TRIM20/MEFV in MDM, which was not detectable in unstimulated cells.

As expected, the expression pattern of TRIM genes following IC-stimulation and activation of MDM through FcγR was completely different from what was observed with IFN. First of all, none of the IFN-induced positive controls were up-regulated. On the contrary, STAT1, OAS2 and APOBEC3F expression was found to be down-regulated by more than 2-fold. As expected, the expression of CSF1 was highly up-regulated in IC-stimulated MDM. Regarding TRIM genes, only 2 were significantly up-regulated: TRIM9 (3.9 fold) and TRIM54 (3.4 fold), whereas 8 of them were down-regulated (TRIM22, 32, 34, 44, 47, 58, 59 and 66).

**Figure 4:**
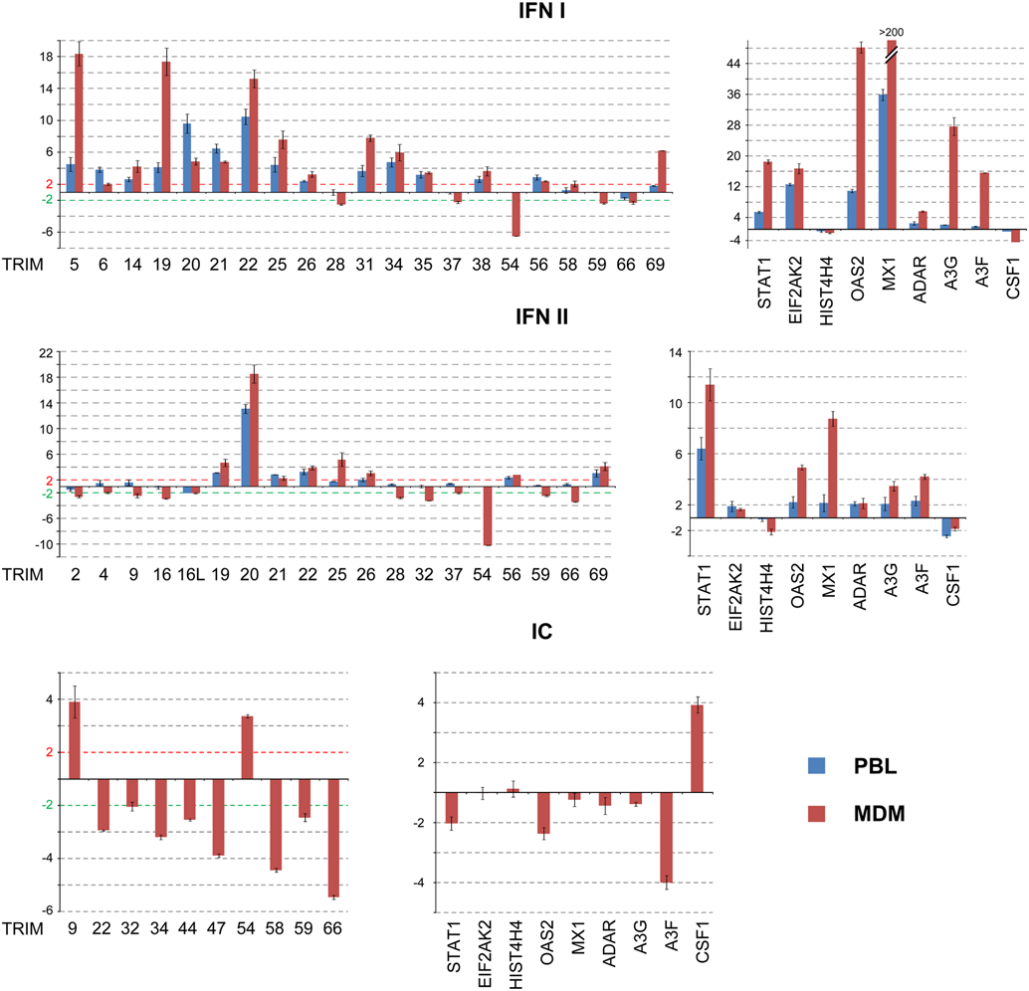
TRIM genes whose expression is regulated by IFN or immune complex. These diagrams show the TRIM genes whose expression is either up- (>2-fold increase compared to untreated cells) or down-regulated (>2-fold decrease compared to untreated cells) by type I IFN, type II IFN or Immune complex (IC). The effect of each treatment on the expression of non-TRIM genes included in the screen is also shown. Data are represented as fold induction.

Quantitative data on the regulation of TRIM genes whose expression was significantly affected by type I or type II IFN in PBL or MDM or by IC in MDM is represented in Figure 4. It must be noted that IFN-treated MDM and PBL gave overall similar profiles, but variations of gene expression were usually more pronounced in MDM. This probably reflects their higher sensitivity to IFN. Among the 16 TRIM genes up-regulated by type I IFN, only TRIM5, TRIM19/PML and TRIM22 showed a >10-fold induction in MDM. TRIM genes were less susceptible to type II IFN, since only TRIM19/PML, TRIM22 and TRIM25 were up-regulated approximately 5-fold and MEFV/TRIM20 18-fold. TRIM54 was significantly down-regulated both by type I and type II IFN, 6 and 10-fold, respectively, as opposed to its up-regulation in IC-stimulated MDM.

## Discussion

Our screen revealed that, 45 out of the 72 human TRIM genes show detectable *ex vivo* expression in blood lymphocytes or unstimulated MDM. Upon type I IFN treatment, 3 additional TRIM transcripts can be detected in both cell types. Twenty seven human TRIM genes were found to be sensitive to IFN treatment, among which 16 were up-regulated by type I IFN and 8 by type II IFN.

**Figure 5:**
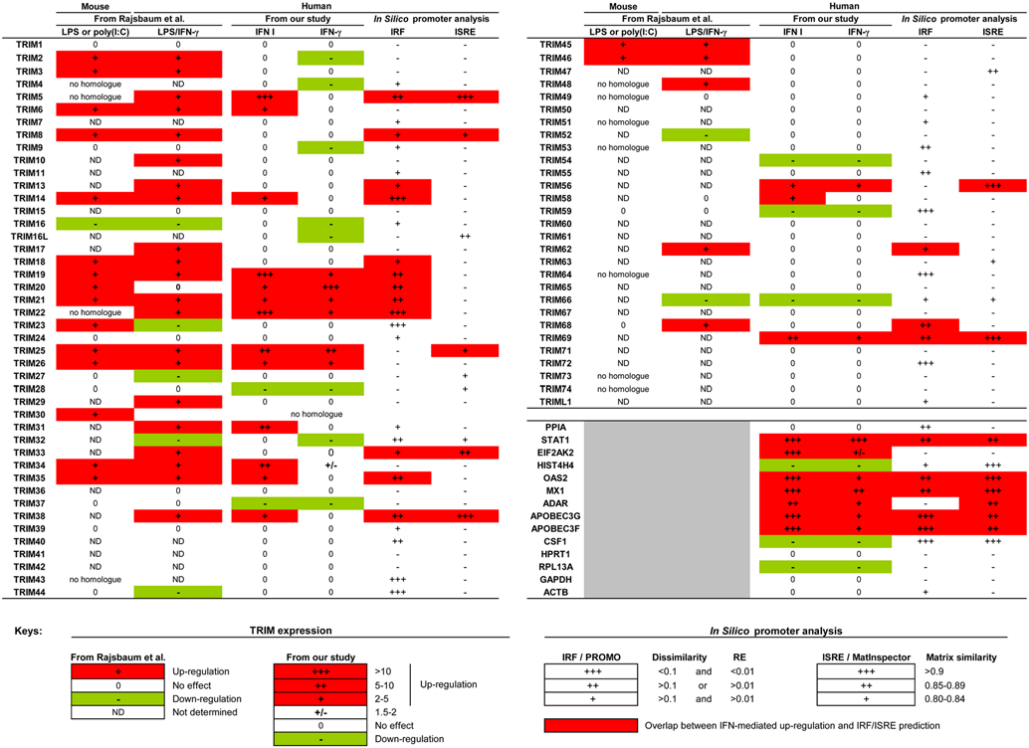
Summary of TRIM expression in mouse and human macrophages upon various stimuli and *in silico* promoter analysis. TRIM expression in human macrophages upon IFN treatment. This part of the table shows the comparison of TRIM gene expression in mouse macrophages treated with LPS or poly(I:C) [35], in human macrophages upon IFN-γ and LPS treatment [35], [47], and in human macrophages stimulated with either type I or type II (γ) IFN (our study). *In silico* promoter analysis. Table illustrating potential transcription factor binding sites based on sequence analysis of 1 kb of genomic DNA upstream of each TRIM protein. IRF sites were scored using the PROMO virtual laboratory using matrices specific to selected human transcription factors (TFs). Highest scoring TF binding sites (+++) had dissimilarity values of less than 0.1 and random expectation values (noted RE within the table key) of less than 0.01. Calculated sites meeting only one of the above criteria (++) or neither (+) are indicated. ISRE/IRF sites were further corroborated with MatInspector. Highest scoring TF binding sites (+++) had similarity values above 0.9, (++) values between 0.85–0.89, and (+) values between 0.80–0.84. Numerous positive genomic controls (OAS2, MX1, STAT1, APOBEC3G, APOBEC3F, etc.) and their calculated TF profiles were used to evaluate the stringency of hits. Negative genomic controls GAPDH and ACTB were used to evaluate the stringency of the programs.

Interestingly, our data partially overlap with a recent study performed on mouse TRIM genes [35] (Figure 5). For instance, TRIM6, 14, 19/PML, 20/MEFV, 21, 25, 26 and 34 were up-regulated in response to IFNs in our study and classified to cluster 2 or 3 by Rajsbaum et al. These two clusters comprise TRIM genes found to be highly expressed in macrophages and dendritic cells (DC) and whose expression is further induced following influenza virus infection in an IFN-dependent manner [35]. We also identified human TRIM5, 22, 31, 38, 56, 58 or 69 as additional genes induced by type I IFN, but these genes were not analyzed or have no homologue in mouse [35]. Our two studies also show that both constitutive expression and IFN-inducibility of TRIM genes are cell type dependent, which may have an impact on the antiviral properties of individual TRIM family members [35]. Our data also largely confirm a gene expression study performed by Martinez and colleagues [47] and re-analyzed by Rajsbaum et al. in order to examine TRIM gene expression in human macrophages stimulated with IFN-γ and LPS [35]. Despite different experimental conditions, a good overall agreement can be observed (Figure 5). The main discrepancies concern a number of TRIM genes (including TRIM2, 3, 10, 13, 17, 18, 29, 45, 46, 48 and 62) which were found to be up-regulated by IFN-γ/LPS treatment by Martinez et al., although they did not respond to IFNs in our study. It has to be noted that the study by Martinez et al. was performed on M-CSF-treated MDM, further activated for 18 h with LPS and IFN-γ [47]. In contrast, we avoided the use of exogenous cytokines, such as M-CSF, for differentiating monocytes into macrophages, since it may directly induce the expression of several genes [48], [49] and even influence retroviral replication [50]. In addition, we identified TRIM20/MEFV as being highly up-regulated by both type I and type II IFN, in accordance with another study [26], whereas Martinez et al. did not [35].

Interestingly, our analysis revealed that TRIM genes are more sensitive to type I IFN, which is considered as the “antiviral IFN”, than to type II IFN. Up-regulation of TRIM genes by type I IFN may indicate the presence of interferon-stimulation response elements (ISREs) or closely related interferon regulatory factor elements (IRFEs) in the genomic regions upstream of TRIM genes that serve as docking sites for interferon regulatory factors (IRFs) involved in IFN gene regulation. Briefly, of the nine known mammalian IRFs, IRF-1 and IRF-2 have been extensively studied and are known to bind IRFE sequences (consensus: G(A)AAA^G^/_C_^T^/_C_GAAA^G^/_C_^T^/_C_ ) to activate or inactivate gene expression following type I or II IFN stimulation [51]. IRF3 and IRF7 bind ISRE sequences (consensus: ^A^/_G_NGAAANNGAAACT) [52] to activate gene expression; this has been most notably demonstrated for the IFN-β enhanceosome [53]. To look for regulatory elements that respond to type I IFN, we carried out an *in silico* analysis and identified putative ISREs and IRFEs within the upstream regions of several IFN-induced TRIM genes with matrix similarities exceeding 95% (Figure 5, and Table S1 with exact sequence and positions). We chose to focus on ISREs and IRFEs primarily because of their readily identifiable and conserved consensus sequences as well as their preference for type I IFN stimulated transcription factors. While our findings are no substitute for direct functional data on TRIM gene promoters, the correlation with the IFN stimulated up-regulation of TRIM genes is striking and provides a sound basis for future work on the mechanisms of TRIM gene regulation.

**Figure 6:**
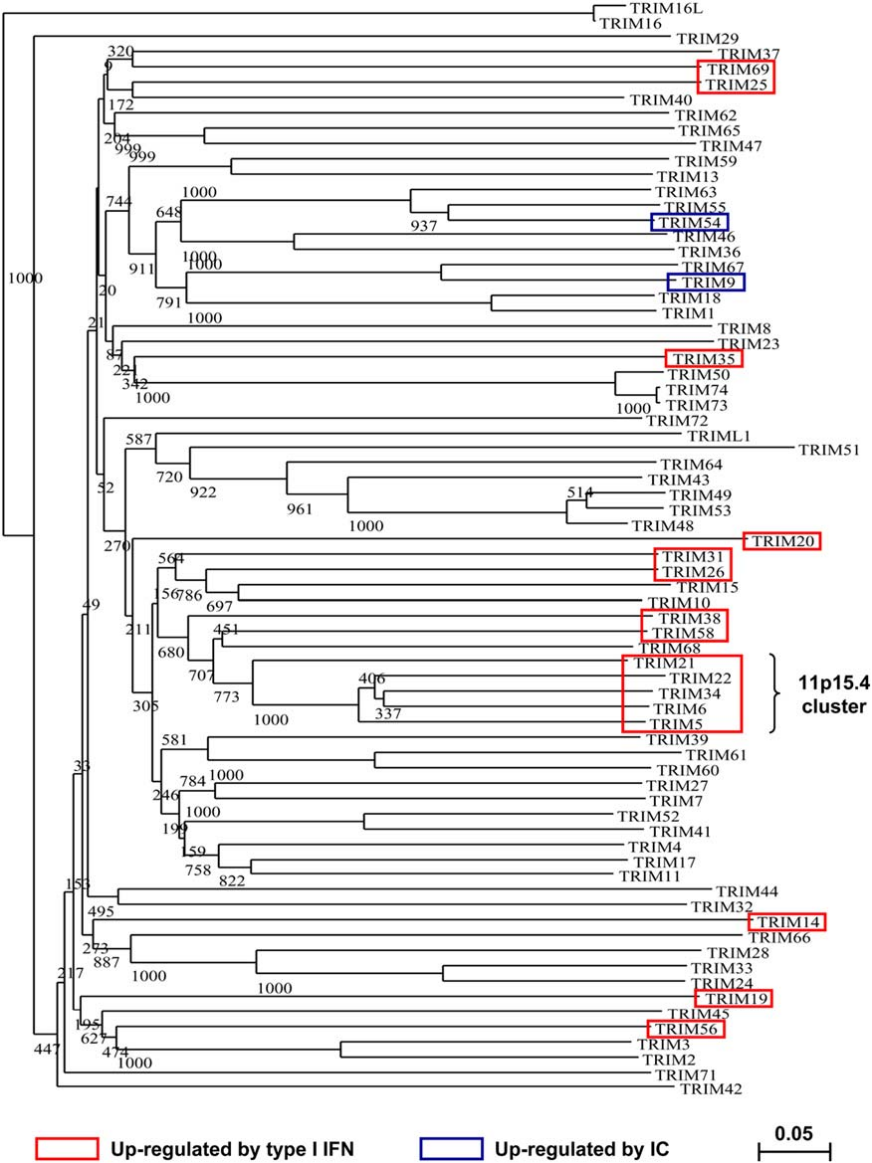
Phylogenetic tree of human TRIM proteins. This joining-neighbor-tree of human TRIM proteins deleted from their Ct domain(s) was constructed using the CLUSTALW and NJplot programs. Numbers indicate bootstrap proportions after 1000 replications. The scale bar represents 0.05 substitutions per amino acid position. Red boxes show TRIM genes which are up-regulated by type I IFN in macrophages, whereas blue boxes show TRIM genes which are up-regulated following activation of MDM with immune complex (IC). TRIM genes belonging to the 11p15.4 cluster are indicated.

There appears to be no correlation between the susceptibility to IFNs and the domain structure. Indeed, TRIM5, 6, 21, 22, 25, 26, 34, 35, 38, 58 and 69 present the RBCC/B30.2 structure characteristic of the C-IV TRIM subfamily, according to the Short and Cox classification [54] (Figure 1), whereas TRIM19/PML, TRIM31 and 56 belong to the C-V subfamily and TRIM14 and TRIM20/MEFV have not been sub-classified, since they lack the RING domain [54] (Figure 1). As shown in Figure 6, TRIM genes whose expression is up-regulated by type I IFN are dispersed throughout the phylogenetic tree of human TRIM genes. The only correlation concerns the TRIM genes localized in the 11p15.4 cluster, which comprise TRIM5, 6, 22 and 34, all of them being up-regulated by IFNs (Figure 6), an observation that has also been reported in murine cells [35]. Two other IFN-induced TRIM genes, TRIM26 (induced by both type I and II IFNs) and TRIM31 (only induced by type I IFN) are located within another major cluster of TRIM encoding genes (containing TRIM10, 15, 26, 31, 39 and 40) located in the major histocompatibility complex region on chromosome 6, at 6p21.33 [55].

Whether induction of gene expression systematically correlates with an increased protein expression will require further studies. Although this is likely, such a correlation may be complicated by the fact that most TRIM genes, if not all, encode several protein isoforms, which may have different sub-cellular localization and stability. Thus, a study on TRIM protein expression would require TRIM isoform-specific antibodies directed against all known members. In consequence, induction by IFNs has been demonstrated at the protein level in the case of very few TRIMs, including TRIM5 [20], TRIM8 [56] and TRIM19/PML [23], [24].

Interestingly, among the 16 TRIM genes we found to be IFN-induced, 10 of them have previously been reported to have an antiviral activity or are involved in processes associated with innate immunity, including TRIM5 [20], [21], [22], TRIM19/PML [23], [24], [25], TRIM20/MEFV [26], TRIM21 [27], [28], TRIM22 [9], [10], TRIM25 [15], TRIM26 [14], TRIM31 [14], TRIM34 [57] and TRIM56 [14]. This observation re-emphasizes the link between IFN-regulation of gene expression and antiviral activity and further suggests a role in antiviral defence for the 6 additional proteins we identified as IFN-inducible (TRIM 6, 14, 35, 38, 58 and 69). Very little is known about these 6 TRIM proteins, apart the fact that TRIM6 can block HIV-1 when its RBCC motif is artificially fused to the B30.2 domain from rhesus macaque TRIM5α [58].

It has to be noted that, although IFN triggers an antiviral response and up-regulates the expression of many TRIM genes, IFN-inducibility is not an absolute prerequisite for displaying antiviral function. Indeed, several TRIM proteins which have been previously involved in innate immunity, such as TRIM1/MID2 [8], TRIM11 [14], TRIM28 [12] or TRIM62 [14], were not found to be induced by IFN. Thus, antiviral TRIM proteins may also be expressed constitutively or be induced through other stimuli, as illustrated by the case of TRIM20 and TRIM35, which were found to be up-regulated following influenza virus infection in murine macrophages and dendritic cells independently of type I IFN receptor expression [35], suggesting the existence of IFN-independent regulation(s) of TRIM expression. In parallel to IFN stimulation, we also investigated the effects of FcγR aggregation by IC on the expression of TRIM proteins. We have indeed shown that IC-stimulation of MDM via the activating FcγR induces the restriction of HIV-1 and related primate lentiviruses after entry [40]. The potential involvement of members of the TRIM family in the restriction should be considered. We were also interested in analyzing the effect of signaling pathways, such as those triggered by FcγR cross-linking by immobilized IC, which can intersect IFN induced Jak-STAT signaling [59], on TRIM gene expression. Interestingly enough, FcγR cross-linking-induced modulation produced a mirror image of IFN induced modulation, down-regulating several TRIM genes induced by IFNs, and up-regulating other genes such as TRIM9 or TRIM54 which were unaffected or highly down-regulated by IFNs, respectively. These last two genes encode TRIM proteins containing a COS box [54], which has been described as a microtubule binding motif [54]. TRIM9 and TRIM54 are only expressed in MDM in the absence of treatment and not in PBL where their expression can only be detected following type II IFN treatment. In mouse primary cells on the contrary, TRIM9 was found to be highly expressed in T-cells but less so or not at all in macrophages and DC [35]. Up-regulation of these two proteins might be related to the MTOC rearrangement in macrophages which is associated to FcγR-triggered phagocytosis [60]. Whether these genes are involved in the induction of an anti-retroviral response in FcγR-activated macrophages warrants further studies.

In conclusion, our study revealed expression modulation of several TRIM genes by two different signaling pathways involved in triggering antiviral responses. Some of these TRIM members have not been previously described as being affected by either IFNs or FcγR engagement. Our results suggest a potential implication of these TRIM proteins in antiviral activities mediated by these stimuli in lymphocytes and MDM. Further functional studies are needed to address this hypothesis.

## Supporting Information

Table S1 In silico identification of IRF binding sites and ISRE within human TRIM gene promoters. Raw data sets for IRF binding sites and ISRE for both PROMO and MatInspector. Numbering is based on the transcription start site at position 1000. PROMO displays TRIMs and potential IRFs as indicated, along with calculated start and end positions of transcription factor (TF) binding sites. Dissimilarity values give the percent difference in sequence similarity between the input TRIM sequence and the calculated TF consensus matrix. RE, or random expectation, yields the probability that the TF consensus binding sequence would occur by chance, where 0.1 denotes 1 occurrence in every 10∧4 bases. MatInspector displays TRIMs, potential IRFs, start and end positions, as well as core and matrix similarities as indicated. Sequences for potential binding sites are shown from both programs.
(0.04 MB XLS)
